# The Little Boy That Died

**Published:** 1877-10

**Authors:** 


### THE LITTLE BOY THAT DIED.

THE late Dr. Chalmers is said to have been the author of the following beautiful lines, written on the occasion of the death of a young son whom he greatly loved:

I AM all alone in my chamber now,
And the midnight hour is near,
And the fagot's crack, and the clock's dull tick,
Are the only sounds I hear;
And over my soul in its solitude
Sweet feelings of sadness glide;
For my heart and my eyes are full when I think
Of the little boy that died.

I went one night to my father's house—
 Went home to the dear ones all,
And softly I opened the garden-gate,
 And softly the door of the hall.
My mother came out to meet her son—
 She kissed me, and then she sighed;
And her head fell on my neck, and she wept
 For the little boy that died.

I shall miss him when the flowers come
 In the garden where he played;
I shall miss him more by the fireside,
 When the flowers are all decayed;
I shall see his toys and his empty chair,
 And the horse he used to ride,
And they will speak with a silent speech,
 Of the little boy that died.

We shall go home to our Father's house—
 To our Father's house in the skies,
Where the hope of souls shall have no blight,
 Our love no broken ties;
We shall roam on the banks of the river of peace,
 And bathe in its blissful tide;
And one of the joys of life shall be,
 The little boy that died.

